# Development of Infant Reaching Strategies to Tactile Targets on the Face

**DOI:** 10.3389/fpsyg.2019.00009

**Published:** 2019-01-21

**Authors:** Lisa K. Chinn, Claire F. Noonan, Matej Hoffmann, Jeffrey J. Lockman

**Affiliations:** ^1^Department of Psychology, Tulane University, New Orleans, LA, United States; ^2^Department of Cybernetics, Faculty of Electrical Engineering, Czech Technical University in Prague, Prague, Czechia

**Keywords:** reaching, tactile localization, prehension, motor development, multisensory coordination, hand-to-mouth coordination

## Abstract

Infant development of reaching to tactile targets on the skin has been studied little, despite its daily use during adaptive behaviors such as removing foreign stimuli or scratching an itch. We longitudinally examined the development of infant reaching strategies (from just under 2 to 11 months) approximately every other week with a vibrotactile stimulus applied to eight different locations on the face (left/right/center temple, left/right ear, left/right mouth corners, and chin). Successful reaching for the stimulus uses tactile input and proprioception to localize the target and move the hand to it. We studied the developmental progression of reaching and grasping strategies. As infants became older the likelihood of using the hand to reach to the target – versus touching the target with another body part or surface such as the upper arm or chair – increased. For trials where infants reached to the target with the hand, infants also refined their hand postures with age. As infants became older, they made fewer contacts with a closed fist or the dorsal part of the hand and more touches/grasps with the fingers or palm. Results suggest that during the first year infants become able to act more precisely on tactile targets on the face.

## Introduction

The ability to act on one’s own body by reaching to specific locations on the body is critical for many tasks of daily living. Although most individuals reach to body locations automatically and with apparent ease, this act involves a coordinated set of perceptual and motor skills. Reaching to a stimulus on the body uses perceptual inputs including touch, proprioception, and sometimes vision to localize a stimulus and to guide a motor action to that location (Longo et al., 2010; Heed et al., 2015). Even though reaching to the body is performed habitually, most reaching studies to date have focused on extending the hand to objects in external peripersonal space. In contrast, little research has addressed reaching to targets on the body or how this ability develops. Here, we longitudinally examine the motor strategies that infants use across the first year as they reach to and grasp a vibrating target placed at different locations on the face.

### Reaching to External Space Versus the Body

Previous work on reaching during infancy has mainly involved the presentation of objects in peripersonal space, external to the body (e.g., Morange and Bloch, 1996). For example, infants from 9 to 20 weeks can first reach to objects placed in front of the ipsilateral shoulder (100% at 9 weeks), then to the midline (33% at 9 weeks and 93% at 17 weeks), and then to contralateral objects (0% at 9 weeks to 71% at 17 weeks; Provine and Westerman, 1979). By 18–20 weeks all infants studied by Provine and Westerman (1979) made contralateral reaches. Studies have also shown that reaching to an object in external space becomes faster, more efficient, and more direct during the first year (Thelen et al., 1993; Berthier and Keen, 2006; Rönnqvist and Domellöf, 2006; D’Souza et al., 2017; Corbetta et al., 2018). Furthermore, reaching is not limited to stimuli that are perceived visually. Infants are also capable of reaching to auditory targets in external space (Clifton et al., 1991).

In contrast to reaching to targets in external space, much less is known about the development of reaching to tactile targets on the body. How is reaching to tactile stimulation on the skin accomplished? Neural research has shown that somatosensory (tactile and proprioceptive) stimulation leads to activations in the somatosensory cortex of the brain, which has been referred to as the “sensory homunculus” (Penfield and Boldrey, 1937). Infants evidence at least a rudimentary somatotopy in these brain regions. By 2 months hand, foot, and lip stimulation leads to different locations of peak somatosensory-evoked potentials recorded with EEG (Saby et al., 2015; Meltzoff et al., 2018). However, such activation *per se* does not mean that the infant localizes the stimulus in the sense that she can reach to it. For that, the stimulation needs to be associated with other sensorimotor laws or contingencies (O’Regan, 2011; Hoffmann et al., 2017).

Reaching to tactile stimuli may initially also be reflexive and controlled in part by spinal or subcortical circuitry. A wiping/scratch reflex has been demonstrated in frogs (Fukson et al., 1980; Berkinblit et al., 1986) and cats (Tapia et al., 2013). Although the existence of such a reflex is debated in humans (MacKay-Lyons, 2002), we cannot exclude the possibility that early reaches to the face – the mouth region in particular – may be brought about by similar mechanisms. However, even if this were the case early in infancy, we would expect these behaviors to become progressively more complex and voluntary over time.

### Processes of Tactile Localization

To localize a tactile stimulus placed on the skin, somatosensory information is “remapped” to an external reference frame (such as body-centered or gaze-centered) in order for a person to reach to the target (e.g., Medina and Coslett, 2010; Heed et al., 2015). The distinctiveness of these skin-based and external representations of the stimulus location can be demonstrated in crossed-limb paradigms where, for instance, the anatomically left hand is located in the right side of external space. Such conflicts are often examined using the temporal order judgment task (Heed and Azañón, 2014) in which adults are slower at identifying the order of touches when the hands are crossed versus uncrossed. Furthermore, in the first half year, a developmental progression occurs in the response to tactile stimuli in crossed feet postures, suggesting that by 6 months infants are beginning to code the position of the crossed feet with respect to external space (Begum Ali et al., 2015). Neural responses associated with limb mapping in external space continue to develop between 6 and 10 months (Rigato et al., 2014).

The previous lines of research associated with tactile perception and body location have mainly focused on behavioral or neural responses that do not involve direct reaching to tactile targets on the body surface. Less is known, however, about the functional ability to reach to target locations on the body and how this sensorimotor skill becomes refined throughout infancy. One sensorimotor ability that may provide a foundation for reaching to some tactile targets, particularly on the face, is the hand-to-mouth transport system (Lew and Butterworth, 1997). Research suggests that hand-mouth coordination is already evident to some degree in the prenatal period, but becomes more skilled and direct in the months immediately following birth (Rochat et al., 1988; Rochat, 1989; Lew and Butterworth, 1997).

### Reaching and Grasping

Being able to transport the hand to the mouth or more generally, contact a stimulus on the face, is only one element of the reaching act. Successful reaching to a stimulus, whether it is located on the body or in external space, typically involves the coordination of at least two different action systems: reaching and grasping (Jeannerod, 1996). Effective reaching requires individuals not only to extend their hands to the location of a stimulus, but open and orient the hand to prepare to grasp the stimulus. Developmentally, research indicates that the reaching system comes online before the grasping system (Piaget, 1952; Bruner, 1973), reflecting a proximodistal sequence in the development of prehension (Lockman and Ashmead, 1983). In particular, before 4 months, infants develop the ability to extend their hand to the location of an object (Piaget, 1952; Bruner and Koslowski, 1972), but during this period the hand is often fisted when it contacts the object. By 4 months, however, infants begin to open the hand in advance of contacting the object. Likewise, with regard to self-touch, closed hand contacts prevail in the first 2 or 3 months, and open hand contacts begin to increase in frequency between 3 and 5 months (Thomas et al., 2015). Nevertheless, it is important to note that with respect to the goal of the reaching act, research on self-touch where infants spontaneously contact a part of their body with their hand might not be directly comparable to research on reaching, where infants are presented a discrete stimulus to reach to in external space.

### The Current Study

In the present work, we consider the problem of reaching to discrete tactile stimuli on the face. We conducted a longitudinal study during the first year in which we placed vibrating targets, one at a time, at different locations on the infant’s face. Because the targets were not accessible to vision, infants had to execute reaches on the basis of tactile and associated proprioceptive information.

In this work, we addressed two main issues. One centered on the different effector systems available to infants for reaching to stimuli on the body and whether infants privilege different effector systems to contact different areas of the face. Specifically, we asked when does the manual effector system become the dominant mode for contacting stimuli on the face. In principle, other movable parts of the body can be used to contact face stimuli. The tongue has the potential to touch external stimuli located near the mouth. Both the head and shoulder can move to establish contact with stimuli on the lower side of the face or the ears. The manual effector system, however, might embody a more effective means for reaching to face stimuli because of the extent to which the arms can move and the precision afforded by fingers that can grasp. To explore these ideas, we asked to what extent infants recruit other parts of the body (e.g., tongue, head, and shoulders) to contact stimuli on the face. If infants, especially at younger ages, touch targets on the face with effectors other than the arms and hands, this would suggest an early awareness at some level of the affordances of the body for reaching to other parts of the body.

The second set of issues that we focused on centered on the manual effector system alone. Specifically, we asked how does grasping become adapted for reaching to targets on the face. We describe how infants’ hand postures when contacting tactile targets and grips on the targets on the face vary with age. We expected that closed fist contacts would decrease with age, while open handed contacts and grips would increase with age. This prediction would be consistent with the idea that infants’ reaching to the face is becoming more skilled and that infants were attempting to grasp these stimuli, which were not a permanent part of the body. Additionally, we were interested in the possibility that different locations on the face might call forth different hand postures depending upon ease or comfort. Modulation of grip strategies or hand postures based on the location of the target might suggest that infants adjust hand posture according to the demands associated with carrying out the reach.

## Materials and Methods

### Participants

A total of 24 infants (10 female; starting age just under 2 to 6 months) were recruited from local daycares, the psychology department of the University, and family-oriented events in the greater New Orleans area. The racial/ethnic backgrounds of participants were Caucasian (*N* = 16), Black/African American (*N* = 3), more than one race (*N* = 3), American Indian (*N* = 1), and Asian (*N* = 1). Three infants did not complete every study visit (one family moved, one had schedule conflicts arise, one dropped out without providing a reason). These infants are included in the data analyses, which were able to accommodate missing data.

### Materials

During the task, gently vibrating targets were fixed to eight locations on infants’ faces/heads one at a time using double-sided skin-safe tape. The target was a disk shape approximately 1.25 cm in diameter, 0.75 cm in height, and 3.5 grams in weight. Inside the target was a flat coin 3-volt DC 70 mA 12000 RPM micro motor that provided vibration similar to vibrating teething rings or mobile phones. The stimuli were coated with black liquid tape to provide a soft and smooth texture. Each testing session was recorded with two mounted video cameras. The experimenter also recorded target location and target contact success on paper, but coding of data analyzed here was done entirely from the videos.

### Procedure and Design

**FIGURE 1:**
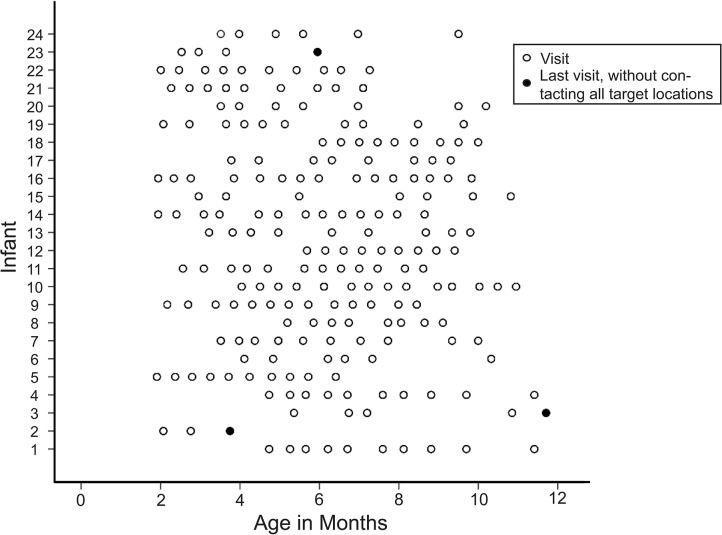
Infant age at each visit with infant ID number randomly assigned. Open circles indicate visits, and closed circles indicate final visits during which the infant did not reach to all target locations (early dropout).

**FIGURE 2:**
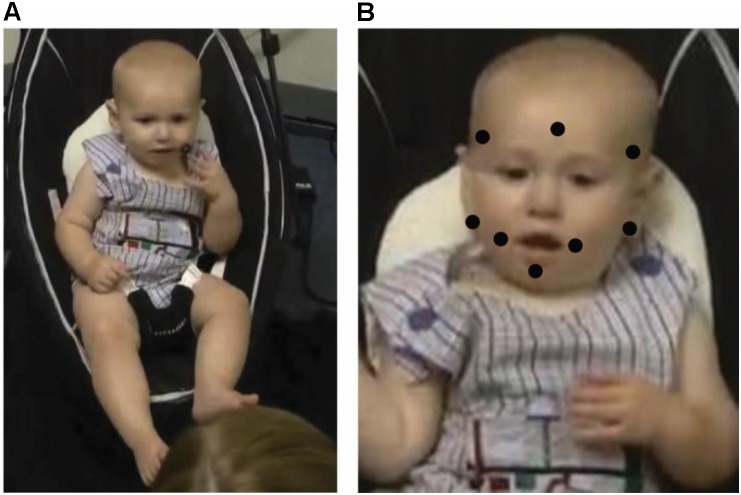
**(A)** Example of infant reaching to target near corner of mouth with ipsilateral hand. **(B)** Diagram of target locations. Written informed parental consent, specifying that a parent approved the use of infant images in written publications, was obtained from a parent of the infant pictured in this figure.

Parents of all subjects provided written informed consent, in which they consented to participating in the study and having the sessions videotaped. They could also choose whether or not to allow images/videos from testing to be used in presentations and written products. The research was approved by the Tulane University Institutional Review Board (reference number 153903) and was consistent with the Declaration of Helsinki. Families were invited to come in for the study every second week until the infant was able to reach to all eight target locations in one visit. Adherence to a schedule with two visits per month was not always possible due to parent schedules or illness (average time between visits = 21.7 days; see Figure 1). The target locations were the left/right corner of the mouth, below the left/right earlobe, on the center of the chin, on the center temple (i.e., forehead), and on the left/right temples (see Figure 2). The order of trials was randomized. When the experimenter applied the lateralized targets, the opposite side of the infant’s face was touched simultaneously at the corresponding location so as not to draw attention to one side of the face over another. For the midline targets, there was no opposite side so the target was placed at midline with no other touch to the face. During each visit, each target was left on the infant’s face until the infant removed it or for approximately 30 s, whichever came first.

Videos of each testing session were coded for factors of target location (chin, left/right mouth, below the left/right ear, and left/right/center temple), whether or not the infants successfully contacted each target (yes, no), how the target was first contacted (left/right hand, left/right arm, head-to-torso, tongue, and head-to-chair), and hand posture when they grasped or contacted the target (closed fist, dorsal hand, palm, finger touch, pincer grasp, and four-finger opposing thumb grasp). When first contact was coded as “head-to-chair,” infants turned the head toward the chair and rubbed the target against the chair. Contacts were not coded as “head-to-chair” if the infant was moving the head in a seemingly random fashion both before and after target placement and then appeared to accidentally graze the chair with the target. In the hand posture coding, the dorsal hand code included only the area on the back of the hand between the wrist and the knuckles; contact with the back of the fingers was coded as finger touch. The palm code included the area of the palm between the wrist and the base of the fingers. Grasps were coded as pincer grips when the index finger and thumb grasped the target.

Here we focus on the development of manual strategies used for successful reaches. In order to accommodate binary outcome variables (e.g., whether the target was first contacted with the hand or not), missing data, and data clustered within each subject, generalized estimating equations (GEEs) were used (See Hardin and Hilbe, 2012). A binomial distribution, a logit link function, and an exchangeable correlation matrix were used. GEEs allow significance testing, while also providing predicted average responses. For example, when a binomial GEE reveals a significant age effect on performance for a measure with a 0/1 scale, it also produces a curve showing predicted average probability of scoring a 1 on the 0/1 scale as age increases.

## Results

### Preliminary Analyses

A primary coder coded 100 percent of the data, and a secondary coder coded an overlapping 20 percent. Inter-rater reliability was achieved for all categorical variables analyzed (mean Cohen’s *k* = 0.87, range = 0.71–1.00). Preliminary analyses found no significant effect of sex or laterality (left versus right target placement and left versus right hand use) on reaching success, so these variables were excluded from further analyses. Further, the age that an infant started the study was not significantly correlated with the age that the infant graduated from the study, indicating that enrolling at a younger age did not result in learning the task earlier.

### How Do Infants Contact Targets?

**FIGURE 3:**
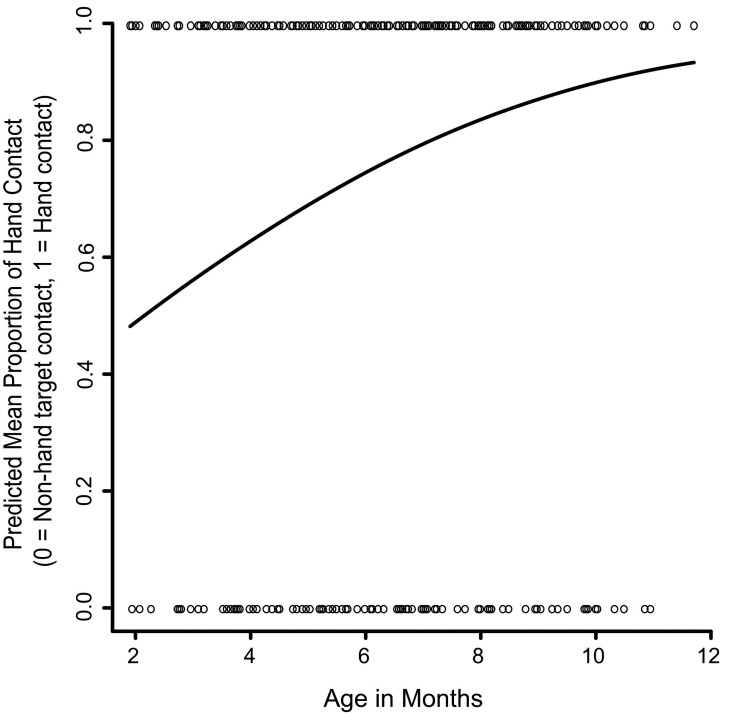
The effect of age on hand versus non-hand contact with the targets. The solid line represents the GEE-predicted probability of hand contact with the targets across age, and the open circles represent the raw data (hand contact or non-hand contact).

**FIGURE 4:**
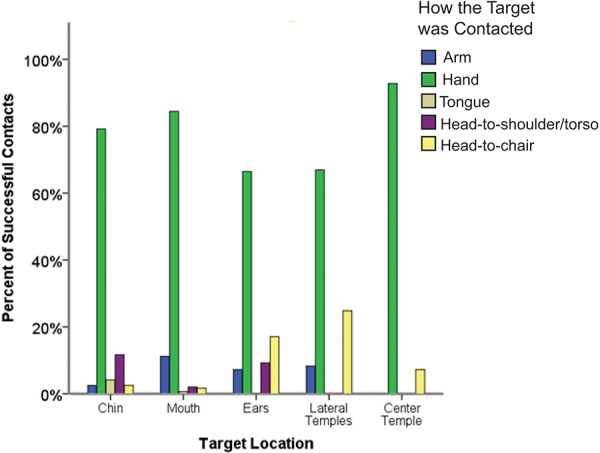
The percentage of successful contacts at each target location with each observed target contact method.

The first set of analyses looked at whether infants chose to use the hand (versus another body part or object) to make contact with the target. Specifically, the first GEE analysis examined the effects of age and target location and an Age x Target Location interaction on hand versus non-hand contact with the target. For 779 out of 1763 total trials (44.19%) infants successfully contacted the target, either using the hand or something else, such as the arm, head-to-torso, tongue, or head-to-chair. Out of 770 of trials where the target contact strategy was visible in our recordings, 599 (77.79%) of these initial contacts were made with the hand (Nine trials that were recorded as successful reaches by the experimenter on paper had a video recording error and are excluded here). As infants became older the likelihood of first contacting the targets directly with the hand versus some other body part or external surface increased significantly (Wald x^2^_1_ = 41.46, *p* < 0.001; Figure 3). Further, the GEE-predicted likelihood of hand contact varied by location (Wald x^2^_4_ = 10.17, *p* < 0.05; Figure 4). GEE predicted the highest percentage of hand contact for the center temple (92%), followed by the mouth (86%), chin (82%), lateral temples (66%), and ears (63%). The Age x Target Location interaction was not statistically significant.

Although the numbers of each type of non-hand contact were too low to analyze statistically (Figure 4), the non-hand strategies used seemed to vary based on the location of the target on the body. Most trials where the head moved to rub the target on the chair involved trials in which the target was placed at the ears or lateral temples and were thus closest to the chair. The head and torso (shoulders or upper chest) came together most often for targets that were placed at the chin or ears – the locations most accessible to the torso. Finally, only mouth and chin targets could be contacted by the tongue, given anatomical constraints of the body.

### Hand Posture and Grips

Next we looked at how hand posture changed with age for trials where infants achieved target contact with the hand. Specifically, we considered the effects of age and target location on whether or not the hand was fisted when it contacted the target, whether the dorsal part of the hand contacted the target, whether the palm/fingers contacted the target, and whether infants used the finger(s) and opposing thumb to grasp the target.

#### Fisted Target Contacts

**FIGURE 5:**
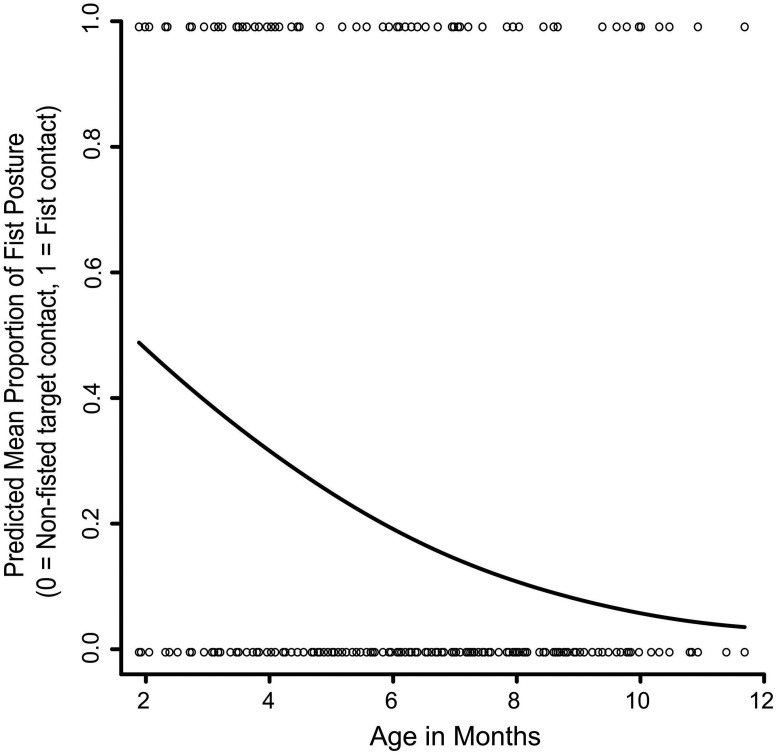
The effect of age on fisted versus non-fisted contact with the targets. The solid line represents the GEE-predicted probability of fisted hand contact with the targets, and the open circles represent the raw data (fisted hand contact or non-fisted hand contact).

The first analysis looked at whether infants became less likely to use a closed hand posture, specifically a closed fist, to contact targets as they became older. Out of 599 trials where initial target contact was made with the hand, 96 contacts (16%) were made with a closed fist. A GEE testing the effects of age, target location, and the Age x Target location interaction revealed that infants became significantly less likely to contact targets with a closed fist as they became older (Wald x^2^_1_ = 28.69, *p* < 0.001; Figure 5). The Age x Target location interaction was statistically significant (Wald x^2^_4_ = 10.26, *p* < 0.05). However, this interaction was difficult to interpret because it largely stemmed from the center temple location, where there were only six fisted target contacts.

#### Dorsal Target Contacts

**FIGURE 6:**
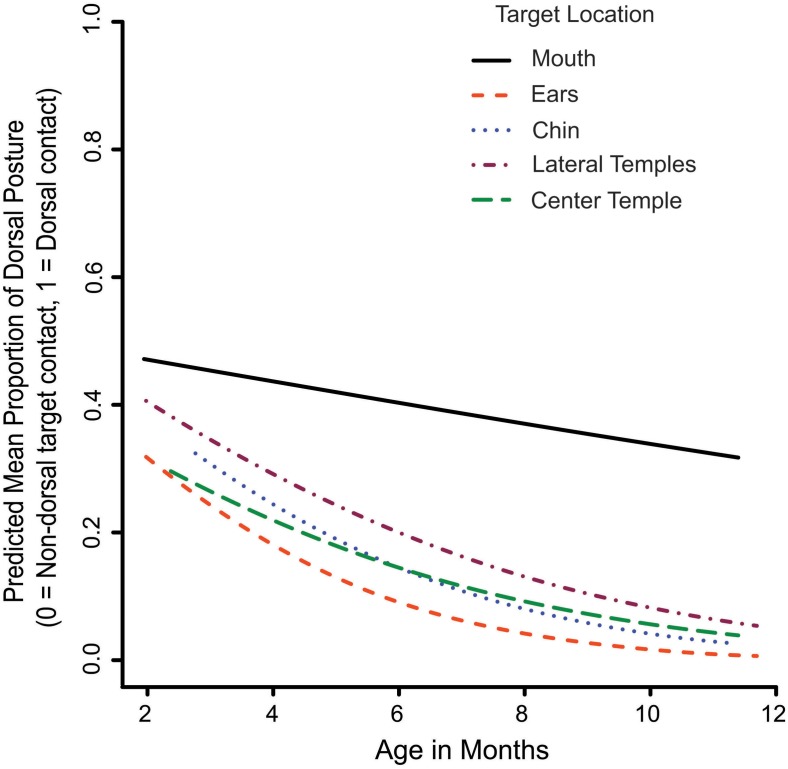
The Age x Target Location interaction on dorsal versus non-dorsal contact with the targets. The lines represent the GEE-predicted probability of dorsal hand contact with the targets for each target location.

The next analysis looked at whether infants became less likely to use the dorsum of the hand to contact targets as they became older, suggesting that infants were attempting instead to touch or grasp the target with the fingers and/or palm. Out of 599 trials where the hand contacted the target, 138 contacts (23%) were with the dorsal part of the hand. A main effect of age showed significantly less dorsal contact as infants became older (Wald x^2^_1_ = 15.03, *p* < 0.001). This main effect, however, was qualified by a significant Age x Target Location interaction on dorsal hand contact (Wald x^2^_4_ = 10.08, *p* < 0.05; Figure 6). Follow up tests showed that dorsal hand contact became significantly less likely at the lateral temples, center temple, ears, and chin (*ps* < 0.01–0.001) as infants became older. For the mouth, age did not significantly affect whether infants used the dorsal part of the hand to contact the target, suggesting that infants may have been attempting another goal with targets located at the mouth.

#### Palm and Fingers

**FIGURE 7:**
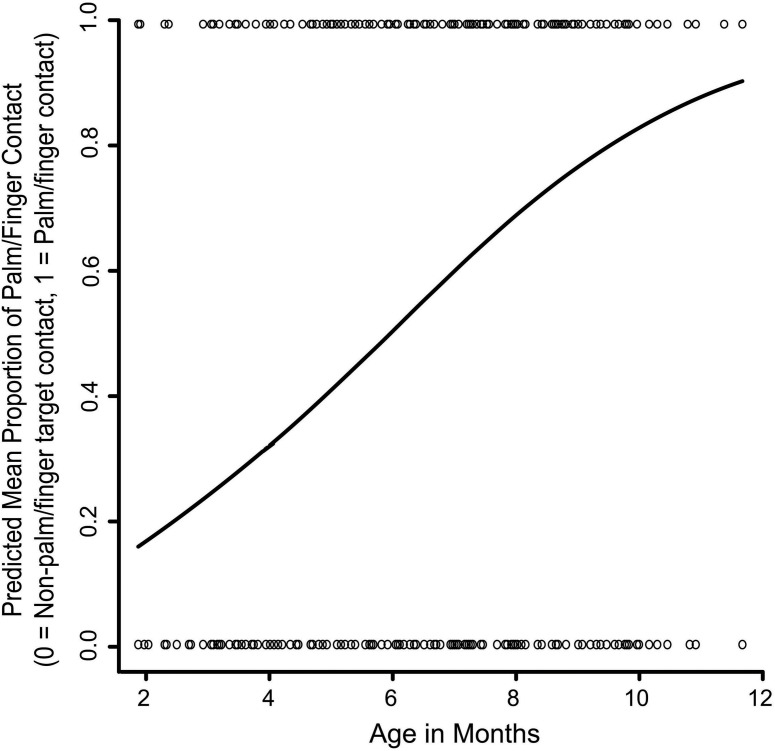
The effect of age on palmar and finger contact versus non-palmar/finger contact with the targets. The solid line represents the GEE-predicted probability of palmar or finger hand contact with the targets, and the open circles represent the raw data (palmar/finger target contact or non-palmar/finger target contact).

In this section, we look at whether palm and finger (ventral or dorsal) contacts versus other forms of contact increase with age. Out of 599 trials where initial target contact was made with the hand 365 (60.93%) contacts used the palm or fingers, and 234 (39.07%) did not use the palm or fingers. As infants became older they were significantly more likely to make contact with the fingers or palm (Wald x^2^_1_ = 40.44, *p* < 0.001; Figure 7). The main effect of target location and the Age x Target Location interaction were not statistically significant.

#### Opposing Thumb Grasps

Next, we looked at whether infants became more likely with age to grasp the target with the opposing thumb and finger(s). Only 63/599 trials (10.52%) involved an opposing thumb grasp, indicating that this strategy was not particularly common in the age range under study. We divided opposing thumb grasps into two different types that we saw infants in this study use - pincer grips (37 trials) and grips with all four fingers opposing the thumb (26 trials). Because there were a limited number of grasp trials, we could only analyze the effect of age in GEE and not the effects of target location and the Age x Target Location interaction. A GEE testing the effect of age on whether the infants used a pincer grip to contact the target showed that pincer grips became more common with age (Wald x^2^_1_ = 24.85, *p* < 0.001). Four finger grips also increased with age (Wald x^2^_1_ = 7.52, *p* < 0.01).

## Discussion

The ability to reach to a source of stimulation on the face is highly adaptive but little studied. Infants reach to stimulation on the face to scratch an itch, but also to remove foreign, potentially dangerous stimuli. Although recent work has shown that the ability to contact vibrotactile targets on the body improves during the first and second years of life (Chinn et al., 2017), this previous work focused on whether infants were able to reach to targets, without examining specific motor strategies through which they do so. Little is known about the motor strategies that infants use to reach to the face. To address this question, we conducted a longitudinal study over the first year in which vibrotactile targets were placed one at a time at different locations on the face. Because the locations of these targets were not accessible to vision, infants had to rely on tactile and proprioceptive information to localize and reach to these targets.

In this study, we found that when a vibrotactile target is applied to the face, infants are more likely to reach to the target with the hand rather than using other effectors or strategies (e.g., rubbing the target on the chair) and that hand versus non-hand use increases with age. They also become more likely to use the palmar surface or fingers of the hand than the dorsum, and they grasp the targets more as they become older. We consider these findings in more detail below.

### Motor Strategies for Target Contact

A primary goal of the current study was to look at the motor strategies that infants used to contact tactile targets on the face. Most studies on reaching to objects in external space focus on the arm and hand. The predominance of the hand and arm in the reaching literature makes sense given that other parts of the body are not configured to grasp targets as well as the hand. However, it is possible to use other body parts or external surfaces to contact a target location on the body.

Here we addressed whether infants contact stimuli on the face with external objects or body parts other than the hand and if these strategies change with age. We found that the hand was used for most reaches throughout the age range under study, but nevertheless and as hypothesized, its use relative to non-hand contact options increased with age. This result suggests that across the first year infants are becoming more likely to reach with the effector best at grasping.

Although the percentages of each type of non-hand contact were low (Figure 4), the non-hand strategies used seemed to vary based on the location of the target and the anatomy of the body. Most trials where infants turned their heads to rub the target on the chair were trials in which the target was placed to the side of the face (ears or lateral temples) and was, therefore, closest to the chair. The head and torso (shoulders/upper chest) came together most often for targets that were placed at the chin or ears – the locations most accessible to the torso. Only mouth and chin targets were contacted by the tongue, which makes sense given anatomical constraints. For tongue contacts of the mouth and chin targets, however, we cannot entirely rule out the possibility that the rooting reflex, although very weak during the age range under study, contributed to this response.

A possible future research direction would be to study localization strategies in infants or children with disorders affecting sensory processing and/or motor skills. For example, children with autism spectrum disorder are sometimes less responsive to tactile stimulation versus neurotypical controls (Tomchek and Dunn, 2007). It is possible that differences in performance on this task for children with known sensory or motor deficits could help our understanding of the processes involved in reaching to vibrotactile targets on the face.

### Reaching to the Body and Arm/Hand Movements

Another main goal of the current study was to look at hand postures and grasping strategies used for target contacts made with the hand. We found that – as predicted – across the first year, infants became less likely to contact targets with a closed fist and with the dorsal part of the hand. Conversely, they became more likely to use the palm or fingers to contact targets, versus the dorsal hand or a closed fist. They also became more likely to grasp the targets with the finger(s) and opposing thumb with age, although this strategy did not predominate by the end of the age range under study. It is known that the pincer grasp for reaching to external objects is beginning to emerge near the end of the first year, consistent with the results of this study on reaching to targets on the face.

More generally, the developmental patterns we saw for hand postures during body reaches are similar to developmental changes in hand posture during reaches to objects in external space (Piaget, 1952; Bruner, 1973). During the first half-year, reaching motions with the arm develop before the ability to open the hand and then grasp an object in external space. These patterns of development also mirror the order of developmental changes in previous findings on hand position during self-touch by Thomas et al. (2015). They found that fist contacts were common in early infancy, followed by an increase in palmar hand contacts, and then followed by a decrease in grasps on the clothing or body parts during self-touch in the first half-year.

Although the order of changes in reaching posture in our study was similar to Thomas et al. (2015) (decreasing dorsal and closed fist, whilst palmar and grasps increased), our results were not identical. Infants in their study appeared to make grasping motions during self-touch at a younger age than the age at which infants in our study grasped discrete vibrotactile targets on the face. For example, at just 20–24 weeks (∼5–6 months), their infants were on average using grasping motions slightly over 15% of the time. At this age infants in our study were still grasping targets less than 10% of the time. One explanation is that because it is uncertain whether spontaneous self-touch is directed toward a specific location, the demands of planning and executing a reach are less than when reaching to a discrete target on the body. Future work could directly test self-touch, reaching to body targets, and reaching to external objects in the same infants to study whether they use different motor strategies during these different types of reaching.

In some instances, hand posture varied based on target location. Specifically, dorsal contacts of targets decreased with age for all locations except the mouth. One explanation is that motions directed toward the mouth may have been more defensive in nature than reaches to other locations on the face. If infants reacted to mouth targets by wanting to rapidly contact them, they may have been more likely to reach to them with a strategy that they were already familiar with (dorsal hand contact) than one that involved orienting and using the fingers for grasping. Future work may use motion tracking to compare the development of reaching speeds for different tactile target locations, in part to determine whether infants are reacting to some tactile stimuli defensively and trying to remove or brush them aside quickly. Kinematic motion tracking is able to measure details of arm movements such as spatial location, acceleration of movement, and velocity in the age range under study (e.g., Ouss et al., 2018). In our current paradigm, motion tracking markers or cables, however, would have interfered with reaching to our targets. In the future, a markerless technology might be used to overcome this challenge and examine reaching trajectories to tactile face targets.

## Conclusion

This study provides new information about the motor strategies infants use to contact stimuli on the face. Our results suggest that early in the first year the hand is already the preferred effector for contacting the face, and it predominates even more as infants become older. At the same time, when infants use non-hand motor strategies to contact face targets, these strategies appear to be based on the location of the targets. For example, targets on the sides of the face, such as near the ears, can be rubbed on the chair or shoulder but cannot be accessed by the tongue. Furthermore, when infants reach with the hand, motor strategies become better adapted for grasping as infants become older. We found that closed fist and dorsal contact decrease with age; palm and finger contact increase with age, and grasping increases with age. These findings on reaching to the face thus support Jeannerod’s (1996) distinction between reaching and grasping as constituting separate but integrated systems that underlie prehension. Finally, the results reported here raise questions regarding the mechanism(s) that underlie developmental changes in effector use and hand posture strategy use when infants contact targets on their faces. One possibility is that these changes are driven in part by experience associated with reaching to the face. For instance, selection of the hand to reach to the face might be reinforced because the hand can manipulate and explore objects better than other effectors. Likewise, opening the hand during reaching, although in part driven by central nervous system and maturational changes, may also be influenced by experience. Although we cannot easily vary experiential input to infants, often because of ethical issues, studies that use modeling with artificial agents in which input is systematically varied might help to provide answers to these questions (Hoffmann et al., 2017).

## Author Contributions

LC, MH, JL, and CN conceptualized the questions addressed in this study. JL and CN designed the study procedure. LC and CN performed the data collection. LC, MH, and JL conceptualized the statistical models. LC performed the data analysis and made figures under the guidance of JL. LC, MH, and JL drafted the manuscript. CN provided revisions.

## Conflict of Interest Statement

The authors declare that the research was conducted in the absence of any commercial or financial relationships that could be construed as a potential conflict of interest.
